# Weaning Time Affects the Archaeal Community Structure and Functional Potential in Pigs

**DOI:** 10.3389/fmicb.2022.845621

**Published:** 2022-03-21

**Authors:** Feilong Deng, Yunjuan Peng, Zhihao Zhang, Samantha Howe, Zhuosui Wu, Jieying Dou, Yuling Li, Xiaoyuan Wei, Xiaofan Wang, Yong Liang, Jiangchao Zhao, Ying Li

**Affiliations:** ^1^Guangdong Provincial Key Laboratory of Animal Molecular Design and Precise Breeding, College of Life Science and Engineering, Foshan University, Foshan, China; ^2^School of Life Science and Engineering, Foshan University, Foshan, China; ^3^Division of Agriculture, Department of Animal Science, University of Arkansas, Fayetteville, AR, United States; ^4^Institute of Systems Engineering, Macau University of Science and Technology, Macau, China; ^5^The Peng Cheng Laboratory, Shenzhen, China

**Keywords:** swine, archaea, growth performance, function, KEGG pathway

## Abstract

Archaea are considered a “keystone” of the gut microbiome and are linked with the host’s energy harvest and health. Although a few studies have investigated the gut archaea in pigs, especially piglets, little is known about the effects of weaning on archaeal structure and function. In this study, we explored the effects of weaning on the longitudinal changes of archaeal composition, diversity, and functional potential in pigs overtime by re-analyzing a recently published metagenomic dataset that included 176 fecal samples collected from commercial pigs on days 7, 14, 21, 28, 35, 70, and 140 after birth. Overall, the richness and diversity of archaeal species showed an increasing trend, and weaning significantly affected the richness of archaeal species. *Methanobrevibacter A smithii* significantly decreased and was replaced by *Methanobrevibacter A sp900769095* within 2 weeks after weaning. For the functional potential, the richness of KEGG KOs increased over time. LEfSe analysis identified 18 KOs, including for example, ko04623 (cytosolic DNA-sensing pathway), ko00500 (starch and sucrose metabolism), and so on, significantly enriched in the weaning pigs, suggesting the involvement of archaea in the piglets’ adaptation to the new diet after weaning. Correlation analysis based on Random Forest regression and Pearson correlation showed that archaeal species richness was significantly associated with pig bodyweight on both days 70 and 140. *Methanobrevibacter A sp900769095* (*R* = 0.405, *p* = 0.040) and *Methanobrevibacter A smithii* (*R* = 0.535, *p* = 0.004) were positively linked with pigs’ bodyweight on days 70 and 140, respectively. Our results revealed the dynamic changes of archaeal diversity and functions and demonstrated the effects of weaning on the gut archaea of pigs, suggesting archaea might play essential roles in swine nutrition, metabolism, and growth performance, especially during the critical weaning process.

## Introduction

Archaea are considered a kingdom independent from bacteria with unique energy sources and metabolic characteristics. Archaea are commonly detected in the digestive tract of humans and animals (Guindo et al., 2021), where some species have host-beneficial characteristics, such as reducing hydrogen (a gut fermentation inhibitor) and trimethylamine-N-oxide (TMAO; a harmful product of gut bacteria). Several studies have reported that the lower feed efficiency of beef cattle and sheep are linked to the enrichment of methane-producing archaea in the rumen (Li and Guan, 2017; Li et al., 2019; McLoughlin et al., 2020). Xue et al. (2020) revealed that specific archaeal species are significantly enriched in high producing dairy cows and milk protein. Given the potential probiotic function of archaea, they have been considered a novel probiotics candidate referred to as “Archaebiotics” (Gaci et al., 2014).

In pigs, archaea compose a small percentage of the gut microbiome, and *Methanobrevibacter* was reported as the dominant genus of archaea in the digestive tract, especially in the hindgut (Mao et al., 2011; Gresse et al., 2019). Su et al. (2014) showed that methanogenic archaea colonized the piglet gut after birth, and in early life, the structure of methanogenic archaea changed dynamically over time according to 16S rRNA gene pyrosequencing. Federici et al. (2015) used PCR-denaturing gradient gel electrophoresis (DGGE) and qPCR methods to detect the archaeal community in piglets and found that weaning affected the archaeal composition of piglets, changing the predominant species from *Methanobrevibacter smithii* to *Methanobrevibacter boviskoreani*. We recently re-analyzed a shotgun metagenomic dataset to investigate the structure and functional potential of archaea in the swine gastrointestinal tract. Our results showed a different archaeal composition and structure compared to other studies. At the same time, our study revealed that the gut archaea in pigs involved in carbohydrate metabolism and hydrogen consumption to affect gut fermentation (Deng et al., 2021). These studies provide essential information regarding archaea in the swine gastrointestinal tract; however, many knowledge gaps still exist. For instance, what roles do archaea play in the swine gut at different growth stages, and does the archaeal structure and function change over the pigs’ lifespan?

Recently, Holman et al. (2021) performed a study exploring the effect of piglet weaning age on the structure and function of the gut microbiome using both large scale shotgun metagenomic sequencing and 16S rRNA-based sequencing methods and revealed that weaning age affects the structure and function of the gut microbiome. Their study provides experimental data that can be analyzed to determine the effects of weaning on gut archaeal structure, diversity, and functional potential in pigs over time. This paper presents the results of our re-analysis of the shotgun metagenomic dataset from Holman et al.’s study.

## Materials and Methods

### Data Collection

Our study was based on a public dataset published by Holman and colleagues. This dataset contains 176 fecal samples collected from 45 piglets that were randomly assigned to three weaning age groups. Metagenomic sequencing was performed on fecal samples collected on days 7, 14, 21, 28, 35, 70, and 140 with the Illumina NovaSeq 6000. Sequences were downloaded from the NCBI SRA database under accession code PRJNA629856. Details of the study design and sample collections were described in the original study (Holman et al., 2021) to explore the effects of weaning on archaea. Complete bodyweight data from different growth stages were provided by Dr. Holman, allowing us to detect the relationship between archaea taxa and growth performance in pigs.

### Raw Reads Pre-processing

First, quality filter and host contamination removal were performed on the downloaded shotgun metagenomic data using the KneadData pipeline (v 0.7.2).^1^ Briefly, raw reads were trimmed based on Phred score using Trimmomatic v0.39 (Bolger et al., 2014). Low-quality regions with a Phred score <20 within a 4 bp sliding window on reads were trimmed and reads shorter than 60 bp were removed. Reads were aligned to the swine reference genome (Scrofa 11.1)^2^ using bmtagger v.3.102.4, and host contamination reads were deleted (Rotmistrovsky and Agarwala, 2011, unpublished). Clean reads of each sample were acquired after the above-mentioned raw sequence data processing steps.

### Archaeal Taxonomy Profiling and Diversity Calculation

Kraken2 (Wood et al., 2019) taxonomic classification software was used to assign clean reads to archaeal reference genomes from the Genome Taxonomy Database release 202 (GTDB; Rinke et al., 2021), which contains archaeal and bacterial genomes from both cultured and uncultured microorganisms. The GTDB contains 4,316 archaeal genomes, representing 2,339 archaeal species and 851 genera, and 254,090 bacterial genomes from 45,555 species (Access date: October 13, 2021), making it a relatively complete reference genome database especially compared to NCBI RefSeq (Haft et al., 2018). The GTDB release 202 was pre-built using the Struo2 pipeline (Youngblut and Ley, 2021) to meet the format requirement of Kraken2. Subsequently, clean reads were assigned to bacterial and archaeal taxa in the GTDB database using Kraken2. Finally, we extracted the reads classified as archaeal reads for further functional analysis. A species-level archaea reads count table was imported into QIIME2 version 2021.4 (Bolyen et al., 2019) to calculate relative abundance, alpha diversity (Shannon index and Observed species), beta diversity (Jaccard and Bray–Curtis), and perform related statistical analyses.

### Metabolic Potential Analysis

To analyze the archaea functional potential, metagenomic clean reads from each sample were assembled separately and co-assembled using MEGAHIT v1.2.9 (Li et al., 2016) software, a total number of 16,300,166 contigs with average length of 2,684 bp ranging from 1,000 bp to 917,796 bp (*N*_50_ = 1,571) were assembled. Gene prediction followed by removal of the redundant genes (allowed similarity < 0.95) were sequentially performed on assembled contigs using Prodigal (Hyatt et al., 2010) and CD-HIT (Fu et al., 2012) to construct a unique gene set. Unique genes were then annotated using eggNOG-mapper (Huerta-Cepas et al., 2019). We quantified the abundance of unique genes by mapping archaea reads to unique gene sequences using Salmon (quant; Patro et al., 2017). Finally, we calculated KEGG pathway KOs abundance using an in-house python script.

### Statistical Analysis

Alpha and beta diversity of archaeal species and potential gene functions (KEGG Orthology) were measured using the QIIME2 platform (Bolyen et al., 2019). Kruskal–Wallis and analysis of similarities (ANOSIM) were applied to calculate the differences in alpha and beta diversity between groups. For all analyses, statistical significance was determined at *p* ≤ 0.05. All figures were generated with the R package ggplot2 (Wickham, 2011).

Regression analysis between the relative abundance of archaea and bodyweights was performed using Random Forest regression in the randomForest R package (Liaw and Wiener, 2002) and Pearson correlation in the R package.

## Results

### Archaeal Alpha and Beta Diversity Were Affected by Age and Weaning

We characterized the dynamics of archaea in the swine gut by re-analyzing 176 fecal samples sequenced using metagenomic technology (Holman et al., 2021). Seven samples contained fewer than 1,672,925 clean reads and were removed after the quality control steps. One sample was considered an outlier and was excluded from further analysis. Finally, an average of 9,006,747 clean reads, ranging from 1,672,925 to 20,672,744, were used for downstream analysis.

We first explored the changes in alpha and beta diversity with age. The overall observed species increased over time throughout all growth stages (from day 7 to day 140), especially throughout the first 28 days, where the observed species of each time point was significantly increased compared to the previous time points (Supplementary Figure S1A). Shannon index first decreased from day 7 to day 21 and then gradually increased from day 21 to day 140 (Supplementary Figure S1B). Additionally, Shannon index was significantly increased on day 28 (*p* = 0.003) and day 70 (*p* = 0.001) compared with the previous time point according to Kruskal–Wallis (pairwise) test.

A significant shift in archaeal community membership (Jaccard distance) between days 7, 14, 21, 28, 35, 70, and 140 were observed according to the PCoA plots (Supplementary Figure S1C). All time points were significantly separated from other groups based on ANOSIM pairwise tests (ANOSIM, *p* ≤ 0.05). However, changes in community membership between days 7, 14, and 21 were greater than the inter-group’s comparison of later neighboring time points according to the ANOSIM *R* value. The *p*-value (lower triangle) and *R* value (upper triangle) of the ANOSIM test based on Jaccard distance are shown in Supplementary Table S1. Similar trends were also observed in the PCoA plot based on Bray–Curtis distance (Supplementary Figure S1D), but no difference was detected between day 28 and day 35 (*R* = 0.039, *p* = 0.102). The *p*-value (lower triangle) and *R* value (upper triangle) of the ANOSIM test of Bray–Curtis distance are shown in Supplementary Table S2.

**Figure 1:**
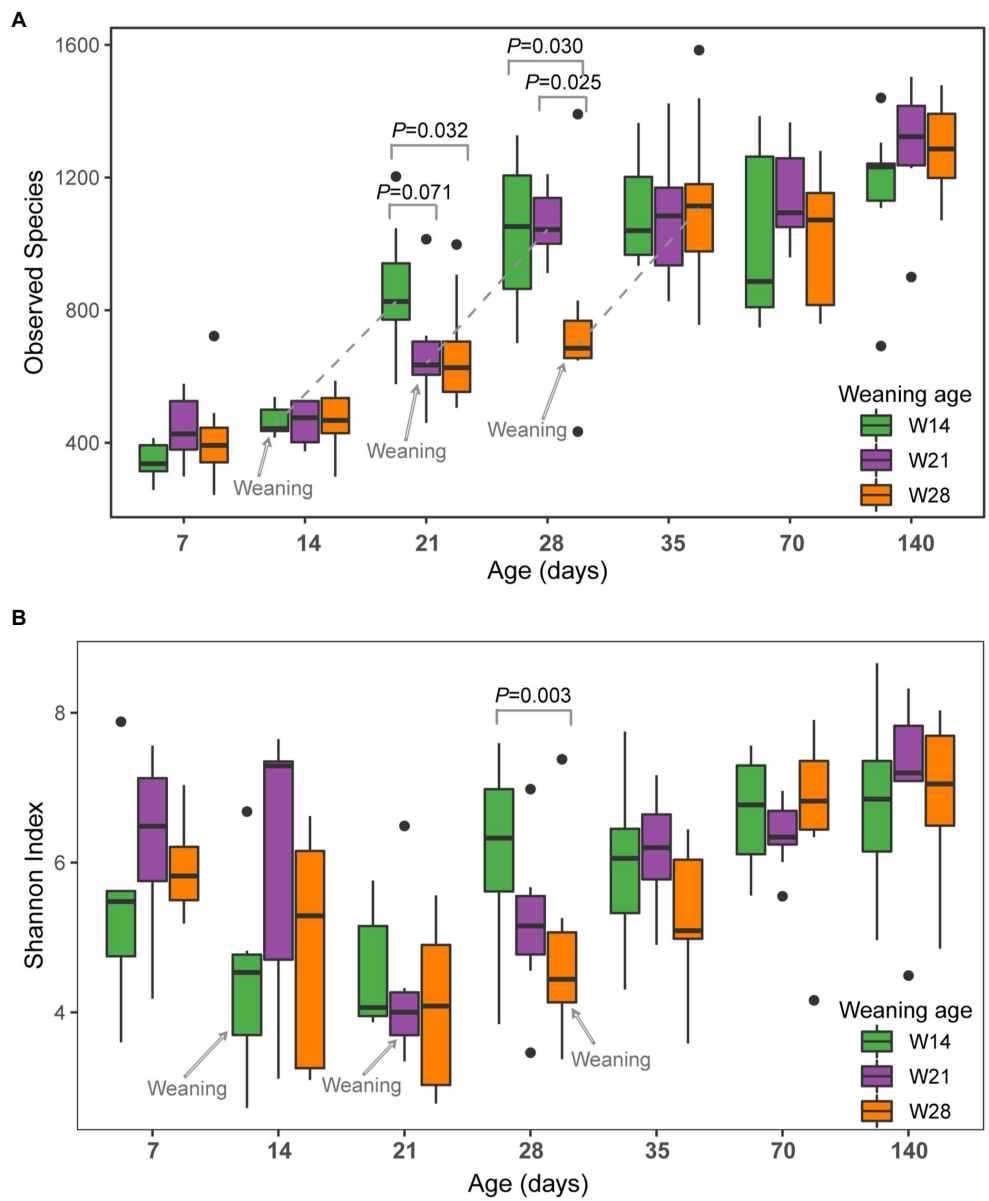
Archaeal alpha diversity in the swine gut grouped by weaning age over time. **(A)** Observed species, **(B)** Shannon Index. Colors of green, purple, and orange represent different weaning time groups on day 14 (W14), day 21 (W21), and day 28 (W28). Significance determined by Kruskal–Wallis (pairwise) test. Values of *p* between groups are labeled above groups when *p* ≤ 0.05. There is no difference between groups (*p* > 0.05) if not indicated otherwise.

During this study, three weaning time points of W14 (Weaning on day 14), W21 (Weaning on day 21), and W28 (Weaning on day 28) were conducted. Therefore, we investigated the effect of weaning time on alpha diversity metrics between the different weaning groups at each time point. A significant difference in observed species was observed between the weaning group and non-weaning groups on days 21 and 28 (Figure 1A). On day 21, the numbers of observed species of W14 (i.e., pigs weaned on day 14) were significantly higher than that of W28 (*p* = 0.032) pigs that were still nursing, as well as the numbers of observed species trend to be higher for W14 than W21 (*p* = 0.071). Additionally, the numbers of observed species of W14 and W21 pigs were significantly higher than those of the W28 group on day 28 (W14 vs. W28, *p* = 0.025; W21 vs. W28, *p* = 0.025). Interestingly, 7 days after weaning (day 35), the archaeal richness of W28 pigs was elevated to a level similar to those of the W14 and W21 groups, and no significant difference was detected between the three groups (*p* > 0.05) on day 35. No trend-specific change was observed for the Shannon index after weaning (Figure 1B), and no significant difference in Shannon index was observed between groups, except between W14 and W28 pigs on day 28 (*p* = 0.003).

### Age and Weaning Together Impact Archaeal Composition

The average relative abundance of total archaea in the gut microbiome (archaea/archaea + bacteria) was 0.62% (ranging from 0.07 to 2.15%). Throughout all growth stages, the lowest relative abundance of total archaea was detected on day 7 (mean = 0.16%). The highest relative abundance of total archaea occurred on day 35 (mean = 0.83%; Supplementary Figure S2). At the genus level, the most abundant genus in the swine gut was *Methanobrevibacter A*, which is defined as a different genus than *Methanobrevibacter* in the GTDB database. The relative abundance of *Methanobrevibacter A* was 0.23% of the total gut microbiome. *Methanobrevibacter* (0.067%), *Methanomethylophilus* (0.062%), and *Methanosphaera* (0.023%) are ranked from second to fourth. *Methanobrevibacter A* is still the dominant genera from day 14 to 140, except for day 7, on which day *Methanobrevibacter* is the dominant genera with a relative abundance of 0.08%.

**Figure 2:**
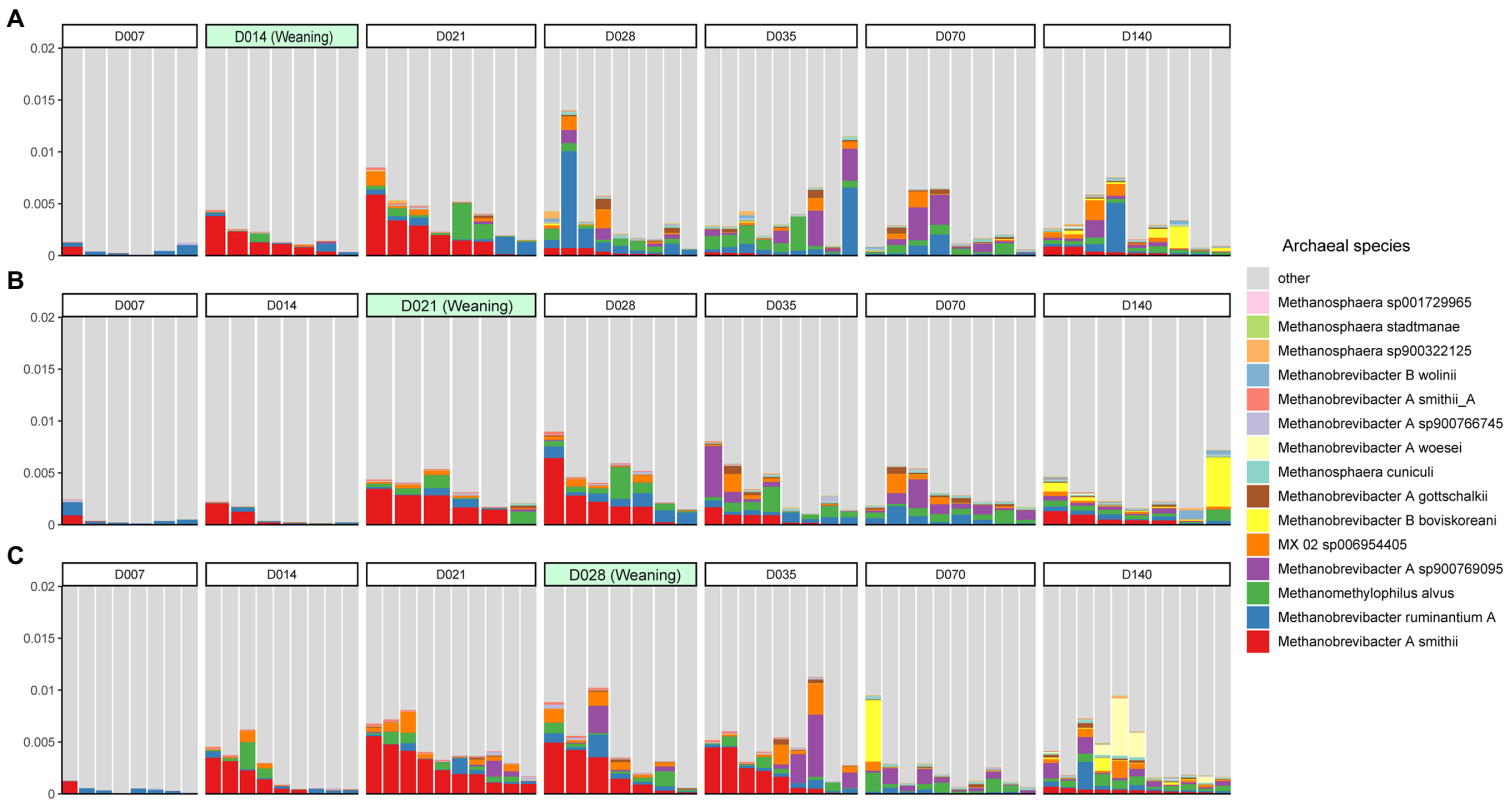
The top 15 most abundant archaeal species in the swine gut during weaning on day 14 **(A)**, day 21 **(B)**, and day 28 **(C)**. Each color represents one archaeal species on the stacked bar chart.

At the species level, *Methanobrevibacter A smithii* (0.11%) was the most abundant species, followed by *Methanobrevibacter ruminantium A* (0.068%), *Methanomethylophilus alvus* (0.063%), and *Methanobrevibacter A sp900769095* (0.051%; Figure 2). However, the dominant species was not the same on all sampling days. *Methanobrevibacter A smithii* was the most abundant species on days 14, 21, and 28, while *Methanobrevibacter A sp900769095* was the most abundant species on days 35 and 70. Additionally, days 7 and 140 shared the same dominant species: *Methanobrevibacter ruminantium A* (Supplementary Figure S3A). Furthermore, the relative abundance of *Methanobrevibacter A smithii* decreased after 14 days of weaning (Supplementary Figures S3B–D).

### Archaeal Functional Potentials in Pigs

**Figure 3:**
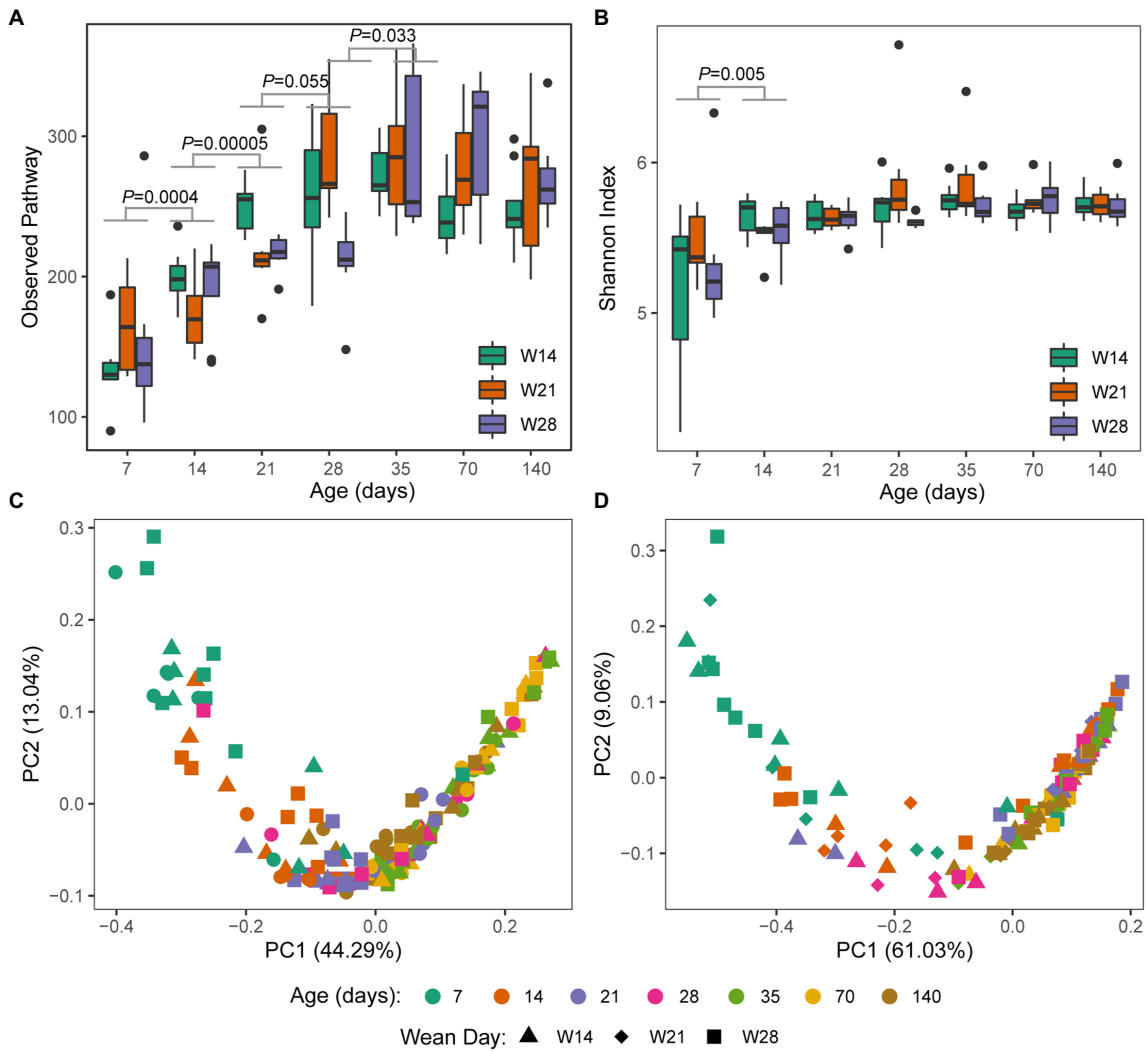
Archaeal functional diversity in swine gut by weaning age over time. **(A)** Observed KEGG Pathways (presence or absence), **(B)** KEGG Shannon diversity, **(C)** PcoA plot based on KEGG pathway membership (Jaccard similarity), and **(D)** PcoA plot based on KEGG pathway structure (Bray–Curtis similarity). Significance determined by Kruskal–Wallis (pairwise) test for both alpha diversity indices (Observed Pathway and Shannon index). Values of *p* between groups are labeled above groups when *p* ≤ 0.05. There is no difference between groups (*p* > 0.05) if not indicated otherwise.

**Figure 4:**
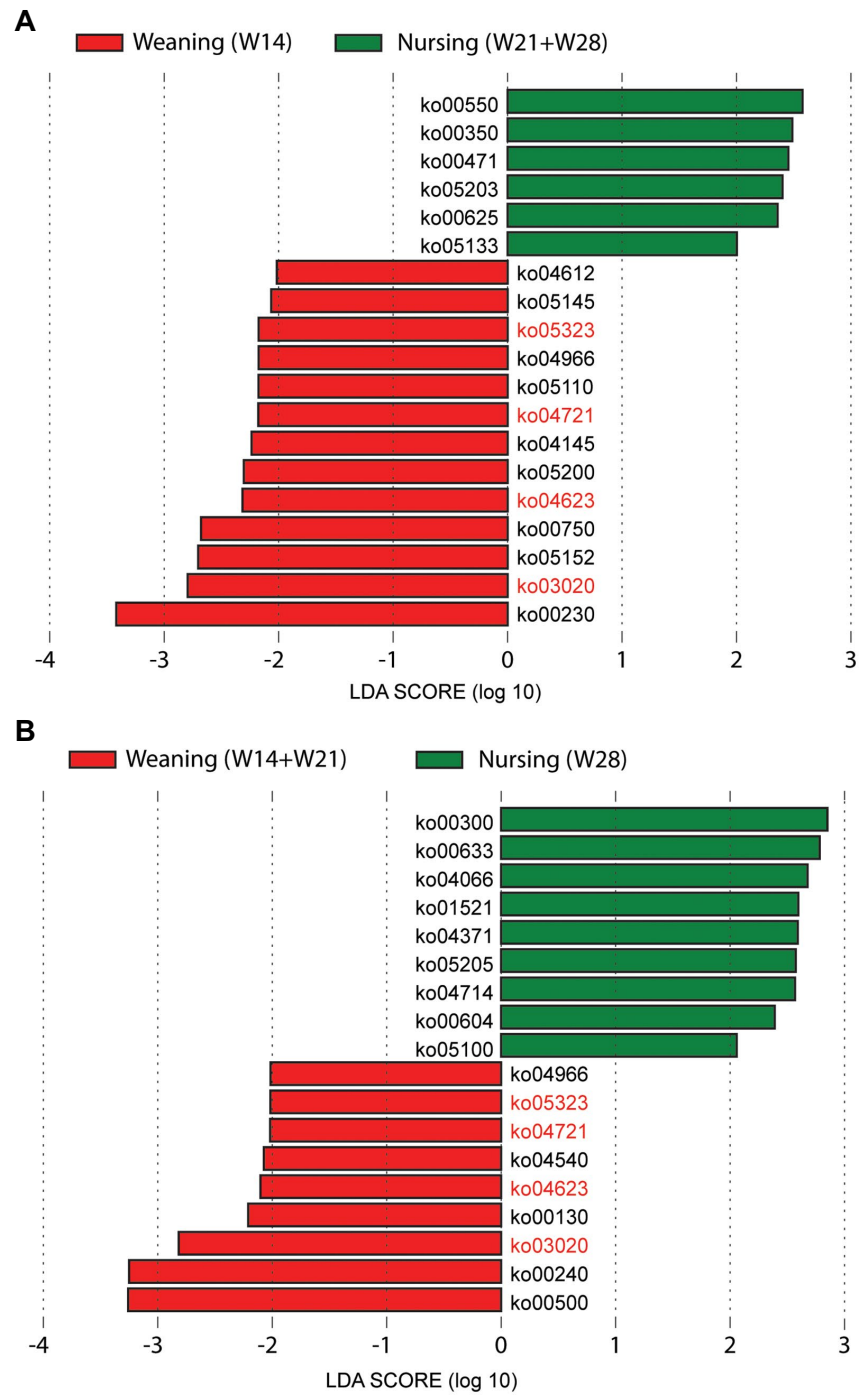
Comparison of archaeal KEGG KOs difference between weaning and nursing group on day 21 **(A)** and 28 **(B)** using LEfSe analysis.

A trend similar to archaeal taxonomy diversity was observed in the KEGG Orthology (KO) diversity of gut archaea (Supplementary Figure S4). Observed KOs increased from day 7 to 35 and then remained stable over time (Figure 3A). The Shannon index of KOs was stable over time, except for a significant increase from day 7 to 14 (Figure 3B). For beta diversity, day 7 was significantly separated from day 14 (ANOSIM, Bray–Curtis: *R* = 0.337, *p* = 0.001; Jaccard: *R* = 0.270, *p* = 0.001) and others for both Jaccard (Figure 3C) and Bray–Curtis (Figure 3D). However, other adjacent time points had a relatively low *R* value based on ANOSIM (*R* < 0.20). Meanwhile, we also found that weaning affects the absence/presence (Jaccard) of KEGG KOs, as weaning and nursing groups were significantly separated on day 21 and day 28 (Supplementary Table S3). However, there was no difference in structure (Bray–Curtis) between the weaning and nursing groups on day 21 (Supplementary Table S4). Significant differences between W14 and W21, both of which were weaned on day 28 (Supplementary Table S4). Additionally, on day 35, there was no (or little, W14 vs. W28, *R* = 0.12) difference of archaeal KEGG KOs absence/presence (Jaccard) between groups (Supplementary Table S3). We further detected the differential KEGG KOs between weaning and nursing groups on days 21 and 28 using LEfSe software (Figure 4). A total number of 18 KOs were significantly enriched in the weaning group on either day 21 or 28 (Table 1). Of them, four KEGG KOs, ko05323 (Immune disease: rheumatoid arthritis), ko04721 (Nervous system: Synaptic vesicle cycle), ko04623 (Immune system: cytosolic DNA-sensing pathway), and ko03020 (Transcription: RNA polymerase), were significantly enriched in the weaning group on both days 21 and 28.

**Table 1:** KEGG KOs enriched in weaning group on day 21 and 28 by the LefSe analysis.

KEGG KOs	Name	Class	Day 21 (Abundance)^*^			Day 28 (Abundance)^*^		
			W14^&^	W21	W28	W14^&^	W21^&^	W28
ko05145	Toxoplasmosis	Human Diseases; Infectious disease: parasitic	7.83	0.00	56.39	95.68	60.99	0.16
ko05323	Rheumatoid arthritis	Human Diseases; Immune disease	110.24	48.06	3.68	187.17	193.53	25.83
ko04966	Collecting duct acid secretion	Organismal Systems; Excretory system	110.24	48.06	3.68	186.27	192.36	1.71
ko05110	Vibrio cholerae infection	Human Diseases; Infectious disease: bacterial	110.24	51.21	311.87	337.26	410.33	161.22
ko04721	Synaptic vesicle cycle	Organismal Systems; Nervous system	114.85	48.06	3.68	186.73	194.47	1.71
ko04145	Phagosome	Cellular Processes; Transport and catabolism	110.24	48.06	310.74	342.26	563.90	146.07
ko05200	Pathways in cancer	Human Diseases; Cancer: overview	45.20	84.55	159.00	208.27	358.07	207.86
ko04623	Cytosolic DNA-sensing pathway	Organismal Systems; Immune system	11.36	0.77	0.00	11.24	95.16	0.00
ko00750	Vitamin B6 metabolism	Metabolism; Metabolism of cofactors and vitamins	405.79	379.01	1,331.09	1,304.35	1,078.32	932.88
ko05152	Tuberculosis	Human Diseases; Infectious disease: bacterial	223.28	346.85	1,063.37	1,246.67	1,121.36	763.25
ko03020	RNA polymerase	Genetic Information Processing; Transcription	1,642.44	1,964.07	5,394.67	5,584.26	4,305.15	4,676.49
ko00230	Purine metabolism	Metabolism; Nucleotide metabolism	5,811.45	7,606.56	20,893.28	20,339.96	18,904.80	19,329.83
ko04966	Collecting duct acid secretion	Organismal Systems; Excretory system	110.24	48.06	3.68	186.27	192.36	1.71
ko04540	Gap junction	Cellular Processes; Cellular community—eukaryotes	0.00	3.63	0.00	57.62	261.92	0.00
ko00130	Ubiquinone and other terpenoid-quinone biosynthesis	Metabolism; Metabolism of cofactors and vitamins	251.32	413.31	953.07	751.19	816.87	1,093.10
ko00240	Pyrimidine metabolism	Metabolism; Nucleotide metabolism	4,847.13	6,814.35	16,952.26	20,428.02	16,472.44	19,896.41
ko00500	Starch and sucrose metabolism	Metabolism; Carbohydrate metabolism	403.06	477.90	975.37	3,909.85	4,445.27	1,335.31

* Average abundance that normalized by the Transcripts Per Kilobase Million (TPM).

& Pigs in this group have been weaned on this day.

### Growth Performance-Associated Archaeal Taxa in Pigs at Different Growth Stages

**Figure 5:**
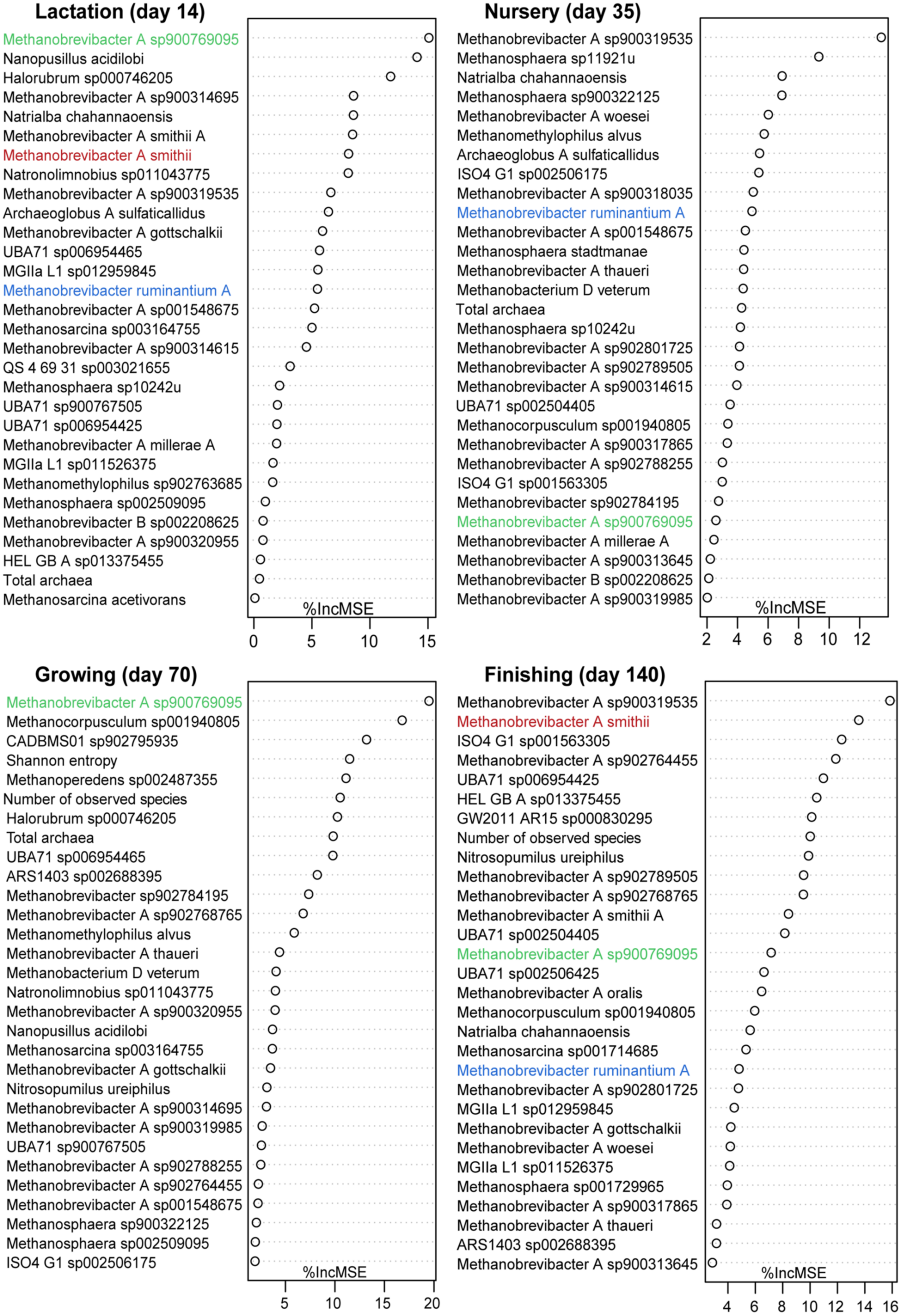
Growth performance-related archaeal species at different growth stages. Top 30 most important bodyweight-related archaeal species on days 14, 35, 70, and 140 were identified using the random forest regression algorithm in R. The most abundant species on different days are highlighted with different colors.

According to previous publications, the archaeal metabolic activity may directly or indirectly contribute to gut fermentation and energy harvest in pigs, meaning that there is potential for archaea to be used as probiotics to improve growth performance. To detect the relation between archaea and bodyweight, regression-based random forest models were developed based on samples collected at days 14, 35, 70, and 140, representing the lactation, nursery, growing, and finishing. Given the relatively low abundance of archaea in the gut, a total number of 73 archaeal species with a relative abundance within the total gut microbiome, defined as total archaea plus total bacteria in this study, higher than 0.05% in at least one sample and both alpha diversity indices (Shannon index and Observed species) were selected for correlation analysis. The top 30 predictors of growth performance at different stages are presented in Figure 5. Most of the top 30 archaeal predictors of bodyweight for each stage belong to the genus *Methanobrevibacter A*. Two uncultured species of *Methanobrevibacter A* were listed as the most important predictor during different stages, *Methanobrevibacter A sp900769095* (days 14 and 70) and *Methanobrevibacter A sp900319535* (days 35 and 140). The number of observed species was listed as one of the top 30 predictors on both days 70 and 140. Furthermore, Pearson’s correlation coefficient analysis showed that observed species was significantly positively correlated with pig bodyweight on both day 70 (*R* = 0.402, *p* = 0.042) and 140 (*R* = 0.541, *p* = 0.004), and no significant association was detected on days 14 and 35 (Supplementary Figure S5). Three species, including *Methanobrevibacter A smithii* (days 7 and 140), *Methanobrevibacter A sp900769095* (days 35 and 70), and *Methanobrevibacter ruminantium A* (days 14, 21, and 28), were the most abundant archaeal species at specific stages; therefore, we chose to focus on them for additional analyses. An association analysis revealed that the relative abundance of *Methanobrevibacter ruminantium A* did not associate with pigs’ bodyweight at any stage. However, a significant association was detected between pigs’ bodyweight on day 140 and the relative abundance of *Methanobrevibacter A smithii* (*R* = 0.535, *p* = 0.004). Interestingly, the relative abundance of *Methanobrevibacter A sp900769095* was significantly correlated with bodyweight on day 70 (*R* = 0.405, *p* = 0.040), at which point its relative abundance increased and replaced *Methanobrevibacter A smithii* as the most abundant archaeal species. However, the correlation was no longer present on day 140 (*R* = 0.012, *p* = 0.951).

To determine if longitudinal changes in archaeal species also correlate growth performance, we developed random forest models to associate changes in archaeal abundances between two time points (day 70 to 140, Supplementary Figure S6A) and bodyweight gain of pigs during this period. Consistent with the random forest models based on the static single time point, *Methanobrevibacter A sp900319535* and *Methanobrevibacter A smithii* were also identified as the top predictors of bodyweight gain by the new model based on dynamic data. Scatter plot shows that *Methanobrevibacter A sp900319535* was significantly positively associated with bodyweight gain from day 70 to 140 (*p* = 0.005, Supplementary Figure S6B). The changes in relative abundance of *Methanobrevibacter A smithii* also had a trend to associate with bodyweight gain from day 70 to 140 (*p* = 0.055, Supplementary Figure S6C).

## Discussion

Several studies have reported that gut archaea may play an essential role as a vital member of the microbiota in humans and animals. The limited number of previous studies have confirmed that, in the mouse model, methanogenic archaea species may be involved in host energy harvest and fat deposition. However, research on gut archaea has been slow to progress due to the inability to effectively culture archaea and their lower relative abundance. Nevertheless, important knowledge could be derived using the available sequencing technologies and bioinformatic tools. In this study, we re-analyzed a shotgun metagenomic dataset previously published by Holman et al. (2021). In doing so, we characterized the dynamic changes of swine gut archaea over time; explored the effects of weaning on gut archaeal diversity, composition, and function; and correlated gut archaea and swine bodyweight at different growth stages. Our study provides important insight into the role of archaea in the swine gastrointestinal tract.

Overall, the relative abundance of total archaea reads increased with age, consistent with what Fernandes et al. (2013) observed in humans. For alpha diversity, an increasing trend of archaeal species (Observed species) colonizing the pig gut was also observed. However, archaeal diversity (Shannon index) first decreased and then increased from day 21 to 140. Fewer archaeal species colonizing the pig gut at an early period but higher equilibrium caused higher Shannon index on days 7 and 14. In humans, the predominant methanogenic archaeal species (*Methanobrevibacter smithii*, *Methanosphaera stadtmanae*, and *Methanomassiliicoccus luminyensis*) significantly changed with age (Dridi et al., 2012). Fernandes et al. (2013) found that total archaea in human fecal samples were significantly positively associated with host age. As far as we know, there is no longitudinal investigation of archaeal diversity throughout the entire production cycle in pigs. In this study, we observed that these pigs’ archaeal richness and diversity is significantly increased on day 140 compared to day 70. However, it should be noted that pigs are still young when slaughtered, and we believe that the gut archaeal diversity had not reached a plateau by day 140 when they were slaughtered, indicating a need for future studies analyzing swine gut archaea later in life.

In addition, we observed that archaeal richness significantly increased following weaning. Federici et al. (2015) reported that weaning piglets elicited a significant shift in the gut archaea population. Chen et al. (2017) found that the gut microbiota diversity of piglets increased significantly in the 10 days after weaning and then reached relative stability, which was also agreed in our results. These results support the point that weaning significantly affects gut archaea in pigs. In our previous study (Wang et al., 2019), we observed an increasing trend in the alpha diversity of the swine gut microbiota over the production period of commercial pigs using 16S rRNA sequencing technology and found that diet was the main driver of the gut bacteria shift, which was observed in other studies (Lu et al., 2018). Moreover, Holman’s study (Holman et al., 2021), the current study’s data source, showed that weaning has a similar effect on gut bacterial diversity, with bacterial richness increasing in the 4 days after weaning. This was revealed by using 16S rRNA sequencing technology on the same batch of samples. These results imply that an archaea–bacteria interaction may cause changes in archaeal and bacterial diversity. However, a recent study of samples from 110 vertebrate species revealed that there is a limited effect of the interactions between archaea and bacteria on archaeal diversity (Youngblut et al., 2021), which means that the change in diet might have independently influenced archaeal and bacterial diversity.

Our study showed that *M. A smithii*, the most abundant species in all samples, was the most affected by weaning as its relative abundance dramatically decreased 7 days after weaning. Federici’s study (Federici et al., 2015) showed that *M. smithii* was abundant on day 28 and then dramatically decreased on day 42 and was undetectable by day 63. At the same time, *M. boviskoreani* increased and replaced *M. smithii* as the dominant archaea species. Given the sensitivity of DGGE, Federici’s results were consistent with ours. In humans, *M. smithii* colonizes the human gut soon after birth and is found in colostrum and breast milk. Additionally, it is widely detected in healthy children and positively associated with organic dairy consumption (Van De Pol et al., 2017). Interestingly, we observed that an unclassified *Methanobrevibacter* species (*M. A sp900769095*) replaced *M. smithii* to become the most abundant species in the growing stage. Except for a metagenomic genome assembly (RefSeq accession number: GCF_900769095.1), no information related to *M. A sp900769095* is available to infer its potential biological function.

We have previously shown that gut archaea have important and unique functional features and are valuable gut microbiome members (Deng et al., 2021). We found that the functional richness of gut archaea in pigs significantly increases at 35 days of age, corresponding to the rapid growth period of archaeal species. Then, archaeal functional richness was stable until the end of sampling on day 140, suggesting that the function of gut archaea developed before day 35 and that functional richness did not increase with the increasing number of archaeal species after that. Additionally, we explored the effects of weaning on archaeal function using LEfSe software and found four KEGG KOs (ko05323, ko04721, ko04623, and ko03020) that were significantly enriched in the weaning group on both days 21 and 28. Ko05323, ko04721, and ko04623 are related to human immune disease (rheumatoid arthritis), nervous system (synaptic vesicle cycle), and immune system (cytosolic DNA-sensing pathway), respectively, suggesting that archaea may respond to weaning stress in piglets. The KEGG pathway of Starch and sucrose metabolism (ko00500) was significantly enriched in the weaning group on day 28, whereas, on day 21, it was enriched, although not significantly, in the weaning group. Starch and sucrose metabolism is essential in gut microbiome carbohydrate metabolism (Zhao et al., 2018). In weaning piglets, the dietary carbohydrate source changes from lactose to plant-based carbohydrates, which require the intestinal microbiota for degradation.

Samuel’s study (Samuel and Gordon, 2006) suggested that hydrogen reduction by *M. smithii* was able to partially, but not entirely, explain the improvement in host energy harvest. Our result suggests that gut archaea potentially enhance the piglets’ ability to extract energy from plant-based carbohydrates.

Random forest regression was used to identify bodyweight-related archaeal species for each growth stage. The Observed species alpha diversity metric was listed as a top predictor for both the growing and finishing stages. Furthermore, Pearson correlation analysis between Observed species and bodyweight confirmed the positive association. Archaea are considered a “keystone” member of the complex microbiome, and their metabolic activity is beneficial for bacteria (Moissl-Eichinger et al., 2018). Here, the increased richness of gut archaea may affect bacteria colonization in the gut, benefitting the pigs. Until now, there have been no reports on the relationship between archaea richness and host growth performance, and further validation of our results is highly warranted.

Both LEfSe analysis and Pearson correlation analysis confirmed that *M. A sp900769095* is positively associated with bodyweight on day 70. Interestingly, at the same time, the relative abundance of *M. A sp900769095* increased after weaning and became the most abundant archaea species on day 35 and day 70, indicating *M. A sp900769095* may play an important role in the growth performance of pigs during the growing stage. Unfortunately, except for the metagenomic genome assembly assembled from a human metagenomic dataset, there is no information over *M. A sp900769095*. It is necessary to culture and further characterize this archaeal species. Additionally, *M. smithii* was positively associated with bodyweight during the finishing stage (day 140). Several previous studies have revealed that *M. smithii* is involved in fat deposition by influencing SCFA production and calorie harvest. Additionally, it is associated with obesity in humans (Samuel and Gordon, 2006; Basseri et al., 2012; Mbakwa et al., 2015). In pigs, the highest rate of body fat deposition occurs during the finishing stage; therefore, the high abundance of *M. smithii* is beneficial during this stage. However, some conflicting results have been reported (Million et al., 2012; Obanda et al., 2018) that suggest that *M. smithii* has no relationship with obesity. In addition, Su et al. reported that lean Yorkshire piglets have a higher abundance of *M. smithii* than obese Meishan piglets. We believe this was likely due to functional differences of *M. smithii* during different growth stages. Nevertheless, additional studies are needed to validate the function of *M. smithii* in pigs.

## Conclusion

Shotgun metagenomic sequencing offers the opportunity to describe the archaeal community structure and functional profile. This study shows the structural and functional changes of swine gut archaea over time and provides new evidence that supports the claim that weaning affects archaeal structure and function. Additionally, we observed the enrichment of many KEGG pathways, especially ko04623, in weaning groups, suggesting that gut archaea respond to weaning stress in piglets. Furthermore, we found that *M. A sp900769095*, an unclassified species, and *M. smithii* are significantly associated with pig bodyweight on days 70 and 140, respectively; however, further validation is necessary.

## Data Availability Statement

The original contributions presented in the study are included in the article/Supplementary Material, further inquiries can be directed to the corresponding authors.

## Author Contributions

FD contributed to analysis and interpretation and drafted the manuscript. YP and ZZ contributed to data analysis. ZW, JD, and YuL contributed to data collection. XWe, XWa, and YoL contributed to interpretation. SH, JZ, and YiL contributed to critically revised the manuscript. JZ and YiL contributed to conception. All authors contributed to the article and approved the submitted version.

## Funding

This work was supported by National Natural Science Foundation of China (No. 32170430), Guangdong Provincial Key Laboratory of Animal Molecular Design and Precise Breeding (2019B030301010), and Key Laboratory of Animal Molecular Design and Precise Breeding of Guangdong Higher Education Institutes (2019KSYS011).

## Conflict of Interest

The authors declare that the research was conducted in the absence of any commercial or financial relationships that could be construed as a potential conflict of interest.

## Publisher’s Note

All claims expressed in this article are solely those of the authors and do not necessarily represent those of their affiliated organizations, or those of the publisher, the editors and the reviewers. Any product that may be evaluated in this article, or claim that may be made by its manufacturer, is not guaranteed or endorsed by the publisher.

## Footnotes

^1^ https://huttenhower.sph.harvard.edu/kneaddata

^2^ ftp://ftp.ncbi.nlm.nirefseq/vertebrate_mammalian/h.gov/genomes/Sus_scrofa/representative/GCF_000003025.6_Sscrofa11.1

## Supplementary Material

SUPPLEMENTARY FIGURE S1 Longitudinal changes of archaeal community diversity and structure in the swine gut with age. Archaeal diversity within samples were measured by Observed species **(A)** and Shannon index **(B)**. Value of *p* based on Kruskal–Wallis (pairwise) tests for Observed species and Shannon index are shown for each neighboring pairwise comparison. The principal coordinate analysis (PCoA) plots based on Jaccard **(C)** and Bray–Curtis **(D)** distance show between-group differences. Different color/shape represent sampling time points from day 7 to day 140.

SUPPLEMENTARY FIGURE S2 Total relative abundances of archaeal reads of the three weaning groups at different growth stages.

SUPPLEMENTARY FIGURE S3 Dynamic changes of the top three archaea species over time in the different weaning groups. *Y*-axes represent the relative abundance of archaea. **(A)** All samples, **(B)** samples weaning on day 14, **(C)** samples weaning on day 21, **(D)** samples weaning on day 28.

SUPPLEMENTARY FIGURE S4 Longitudinal changes of archaeal KEGG KOs diversity and structure in the swine gut. Archaeal diversity within samples were measured by Observed KEGG KOs **(A)** and Shannon index **(B)**. The principal coordinate analysis (PCoA) plots based on Jaccard **(C)** and Bray–Curtis **(D)** distance show between-group differences. Different colors represent sampling time points from day 7 to 140.

SUPPLEMENTARY FIGURE S5 Scatter plots illustrating the association between bodyweight and the four important predictors identified by LefSe analysis. **(A)** Observed species, **(B)** *Methanobrevibacter A sp900769095*, **(C)** *Methanobrevibacter A smithii*, and **(D)** *Methanobrevibacter ruminantium A.* For archaeal species **(B–D)**, *y*-axis represents relative abundance of archaeal species. Pearson’s correlation coefficient (*R*) and Pearson’s correlation test *p*-value are labeled on each plot.

SUPPLEMENTARY FIGURE S6 Bodyweight gain-related archaeal species during growing and finishing stages. The most important bodyweight gain-related archaeal species during finishing stage **(A)**, from day 70 to 140) were identified using the random forest regression algorithm in R. Scatter plots shown the relationship between bodyweight gain and *Methanobrevibacter A sp900319535* **(B)**, and *Methanobrevibacter A smithii* **(C)**. The *y*-axis represents changes of relative abundance of archaeal species, x-axis represents bodyweight gain of pigs from day 70 to 140.
