# Supplementary figures and images for: Weaning Time Affects the Archaeal Community Structure and Functional Potential in Pigs
Source: Front Microbiol. 2022 Mar 21;13:845621. doi: 10.3389/fmicb.2022.845621 (PMC8979004; doi:10.3389/fmicb.2022.845621)

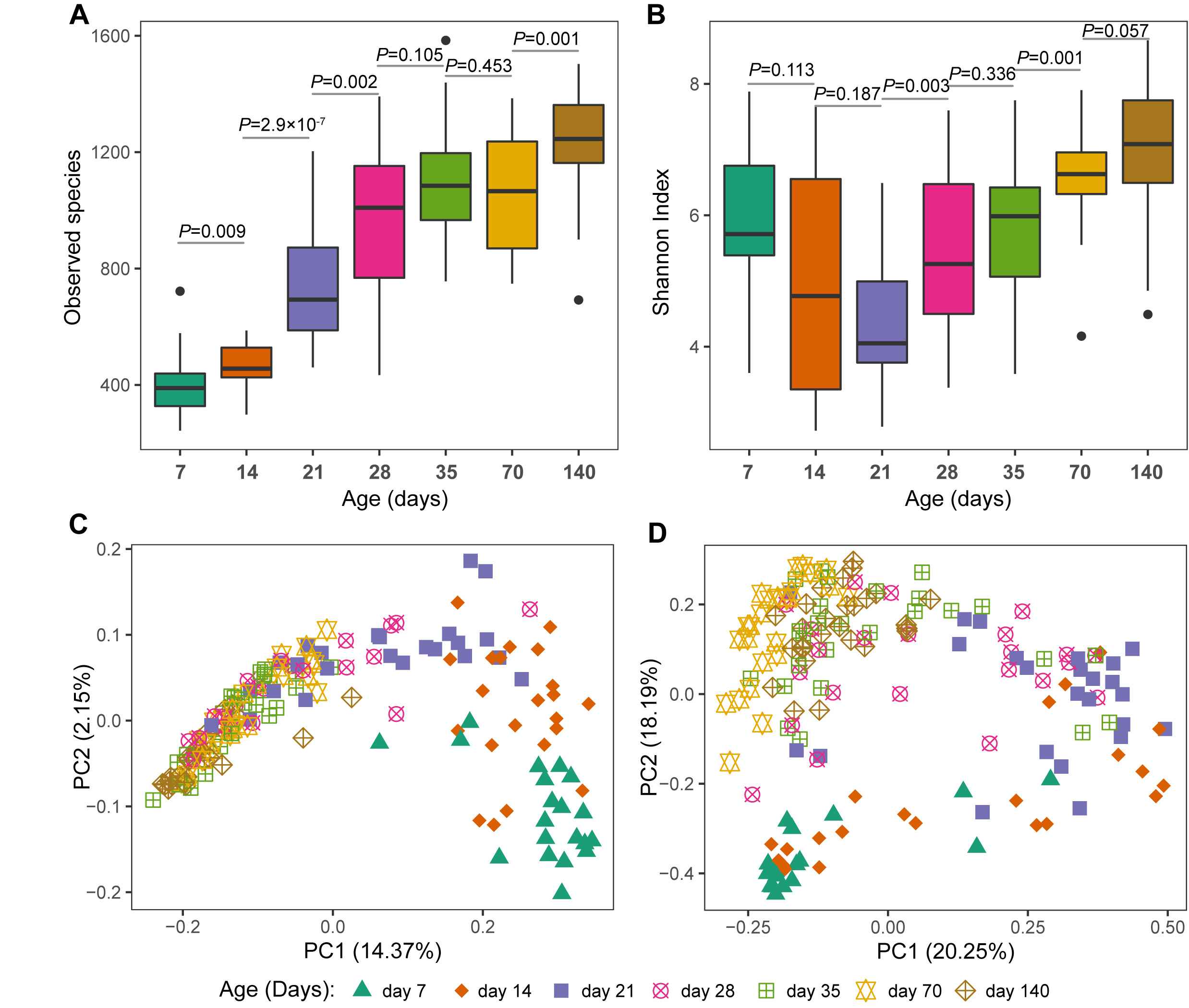

Supplement: SUPPLEMENTARY FIGURE S1 — Longitudinal changes of archaeal community diversity and structure in the swine gut with age. Archaeal diversity within samples were measured by Observed species (A) and Shannon index (B). Value of p based on Kruskal–Wallis (pairwise) tests for Observed species and Shannon index are shown for each neighboring pairwise comparison. The principal coordinate analysis (PCoA) plots based on Jaccard (C) and Bray–Curtis (D) distance show between-group differences. Different color/shape represent sampling time points from day 7 to day 140. [file Image_1.TIF]

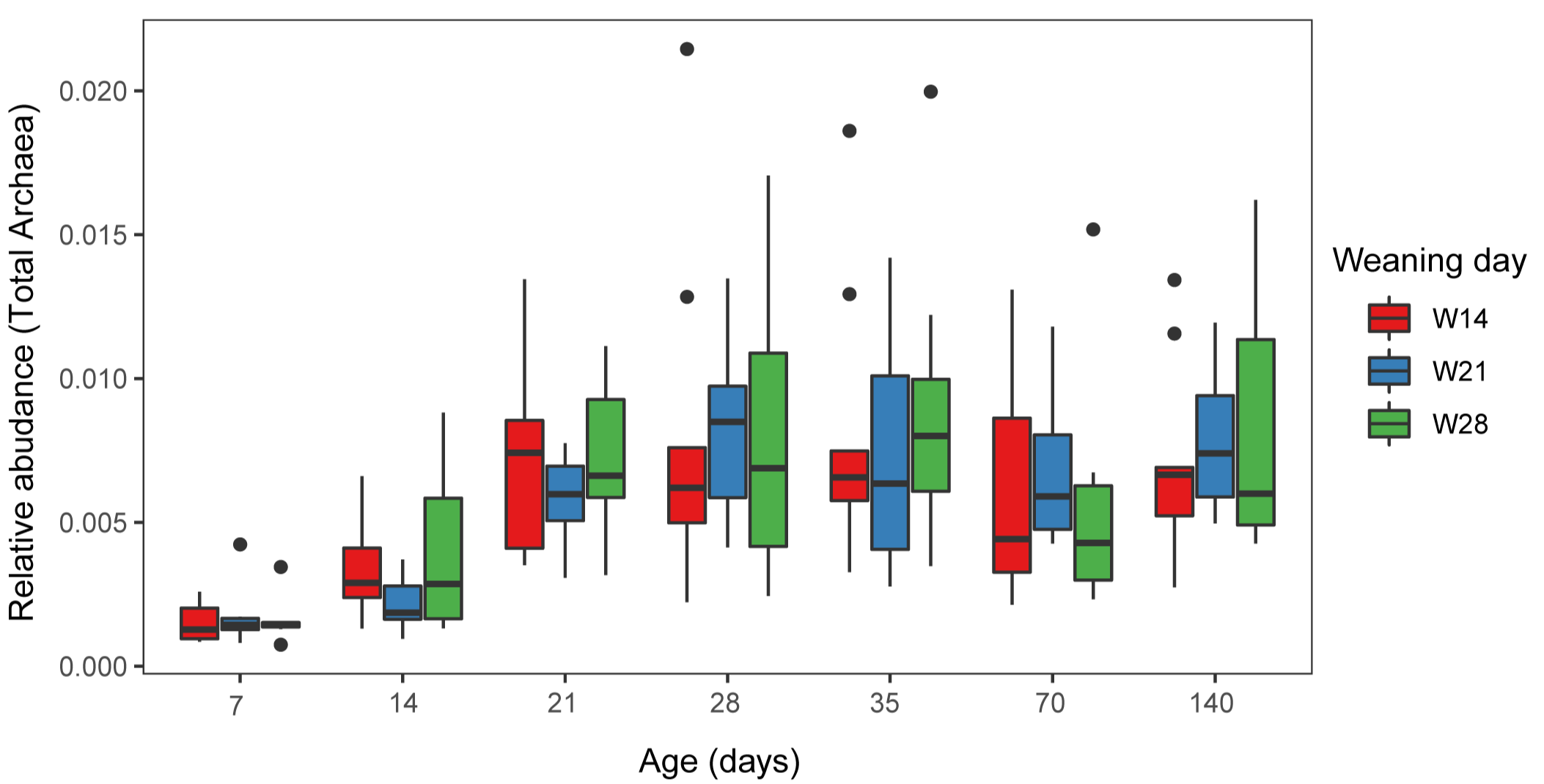

Supplement: SUPPLEMENTARY FIGURE S2 — Total relative abundances of archaeal reads of the three weaning groups at different growth stages. [file Image_2.TIF]

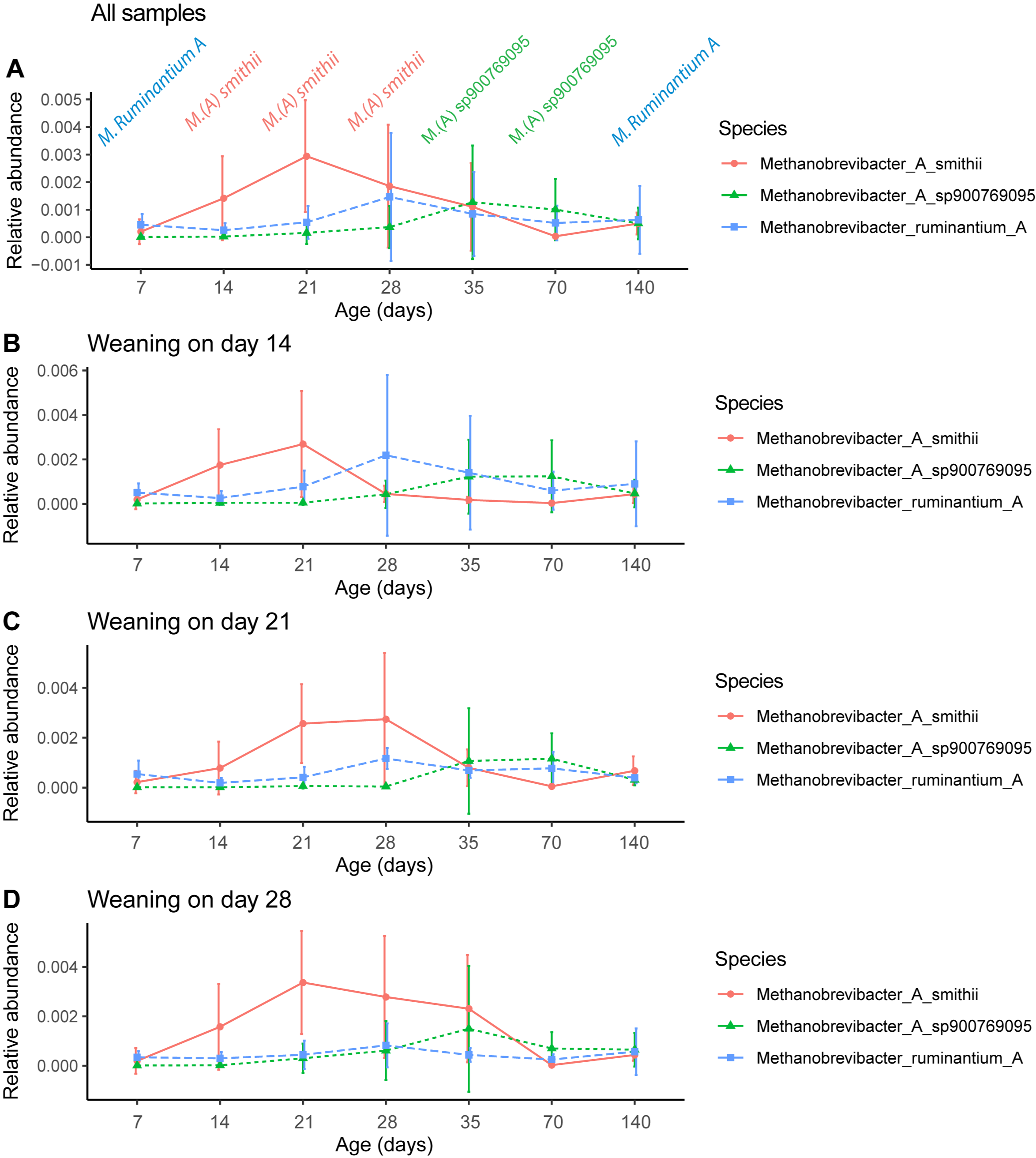

Supplement: SUPPLEMENTARY FIGURE S3 — Dynamic changes of the top three archaea species over time in the different weaning groups. Y-axes represent the relative abundance of archaea. (A) All samples, (B) samples weaning on day 14, (C) samples weaning on day 21, (D) samples weaning on day 28. [file Image_3.TIF]

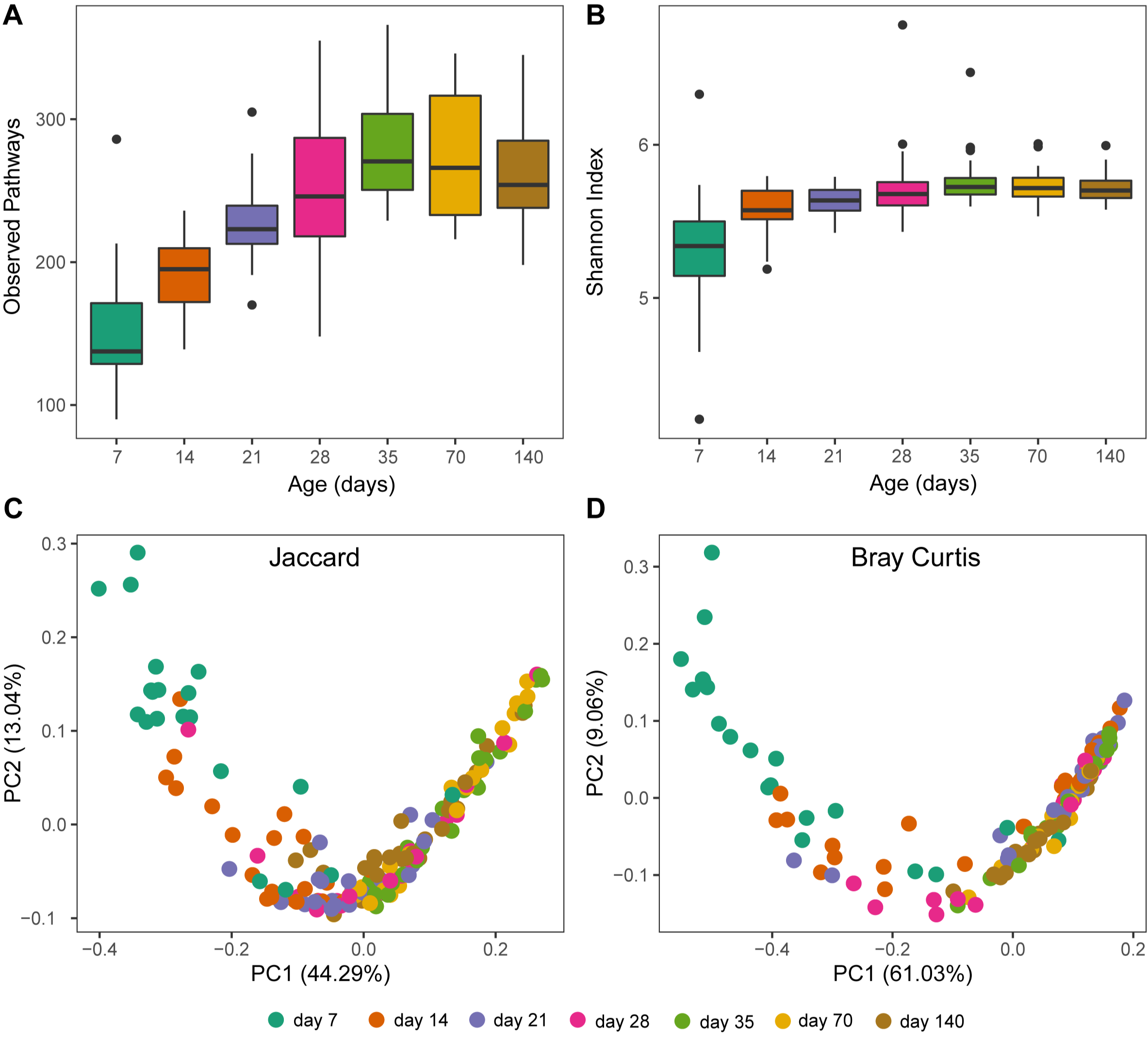

Supplement: SUPPLEMENTARY FIGURE S4 — Longitudinal changes of archaeal KEGG KOs diversity and structure in the swine gut. Archaeal diversity within samples were measured by Observed KEGG KOs (A) and Shannon index (B). The principal coordinate analysis (PCoA) plots based on Jaccard (C) and Bray–Curtis (D) distance show between-group differences. Different colors represent sampling time points from day 7 to 140. [file Image_4.TIF]

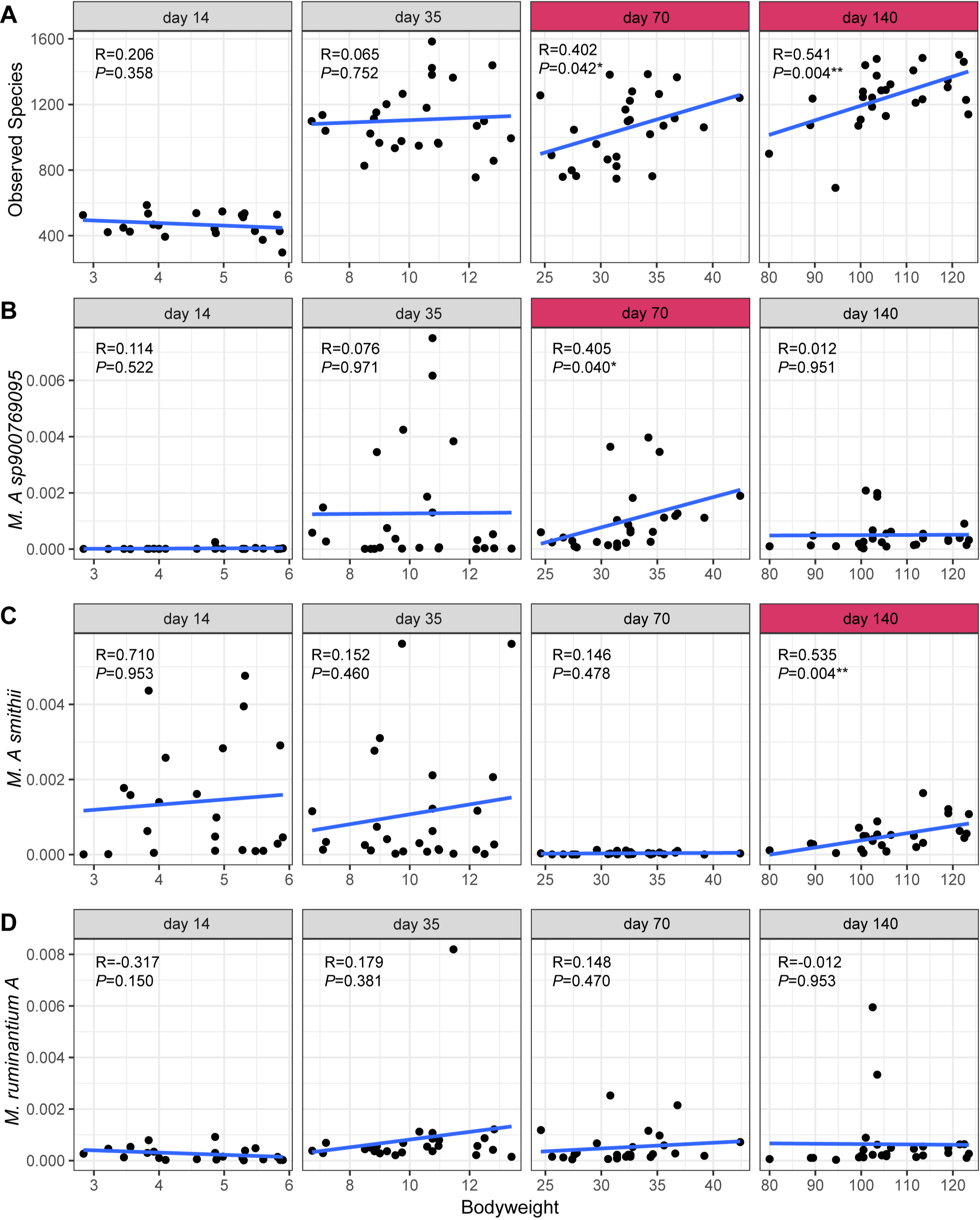

Supplement: SUPPLEMENTARY FIGURE S5 — Scatter plots illustrating the association between bodyweight and the four important predictors identified by LefSe analysis. (A) Observed species, (B) Methanobrevibacter A sp900769095, (C) Methanobrevibacter A smithii, and (D) Methanobrevibacter ruminantium A. For archaeal species (B–D), y-axis represents relative abundance of archaeal species. Pearson’s correlation coefficient (R) and Pearson’s correlation test p-value are labeled on each plot. [file Image_5.TIF]

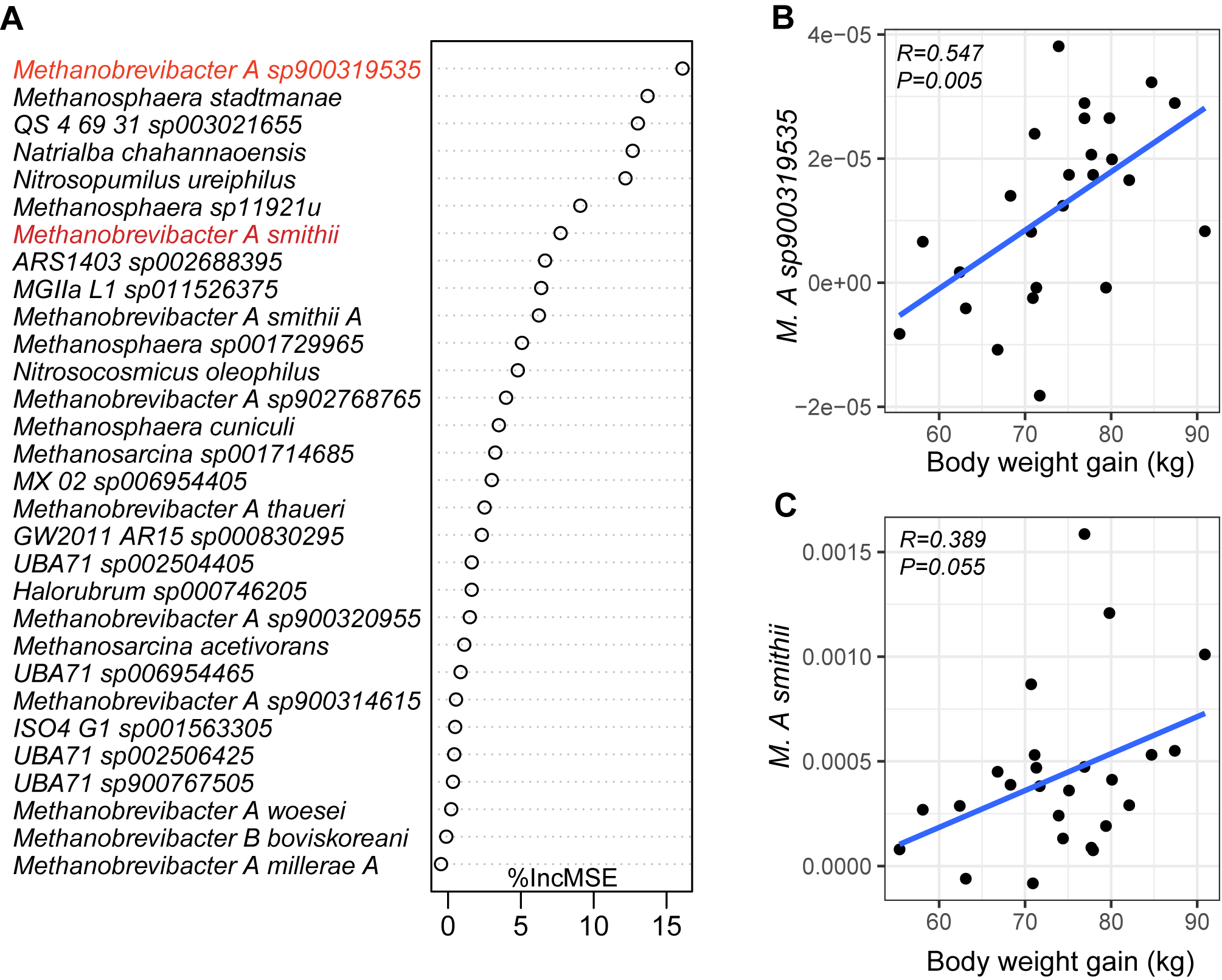

Supplement: SUPPLEMENTARY FIGURE S6 — Bodyweight gain-related archaeal species during growing and finishing stages. The most important bodyweight gain-related archaeal species during finishing stage (A), from day 70 to 140) were identified using the random forest regression algorithm in R. Scatter plots shown the relationship between bodyweight gain and Methanobrevibacter A sp900319535 (B), and Methanobrevibacter A smithii (C). The y-axis represents changes of relative abundance of archaeal species, x-axis represents bodyweight gain of pigs from day 70 to 140. [file Image_6.TIF]
